# Predicting Tumor Mutation Burden and *EGFR* Mutation Using Clinical and Radiomic Features in Patients with Malignant Pulmonary Nodules

**DOI:** 10.3390/jpm13010016

**Published:** 2022-12-22

**Authors:** Wenda Yin, Wei Wang, Chong Zou, Ming Li, Hao Chen, Fanchen Meng, Guozhang Dong, Jie Wang, Qian Yu, Mengting Sun, Lin Xu, Yang Lv, Xiaoxiao Wang, Rong Yin

**Affiliations:** 1 Department of GCP Research Center, Jiangsu Province Hospital of Chinese Medicine, The Affiliated Hospital of Nanjing University of Chinese Medicine, Nanjing 210029, China; 2 Department of Thoracic Surgery, Jiangsu Key Laboratory of Molecular and Translational Cancer Research, Jiangsu Cancer Hospital & Jiangsu Institute of Cancer Research, Nanjing 210009, China; 3 Department of Science and Technology, Jiangsu Cancer Hospital & Jiangsu Institute of Cancer Research, Nanjing 210009, China; 4 Biobank of Lung Cancer, Jiangsu Biobank of Clinical Resources, Nanjing 210009, China; 5 Department of Information Center, Jiangning Hospital, No. 169, Hushan Road, Nanjing 211199, China

**Keywords:** ground glass opacity, prediction model, radiomics, EGFR mutation, tumor mutation burden

## Abstract

Pulmonary nodules (PNs) shown as persistent or growing ground-glass opacities (GGOs) are usually lung adenocarcinomas or their preinvasive lesions. Tumor mutation burden (TMB) and somatic mutations are important determinants for the choice of strategy in patients with lung cancer during therapy. A total of 93 post-operative patients with 108 malignant PNs were enrolled for analysis (75 cases in the training cohort and 33 cases in the validation cohort). Radiomics features were extracted from preoperative non-contrast computed tomography (CT) images of the entire tumor. Using commercial next generation sequencing, we detected TMB status and somatic mutations of all FFPE samples. Here, 870 quantitative radiomics features were extracted from the segmentations of PNs, and pathological and clinical characteristics were collected from medical records. The LASSO (least absolute shrinkage and selection operator) regression and stepwise logistic regressions were performed to establish the predictive model. For the epidermal growth factor receptor (*EGFR*) mutation, the AUCs of the clinical model and the integrative model validated by the validation set were 0.6726 (0.4755–0.8697) and 0.7421 (0.5698–0.9144). For the TMB status, the ROCs showed that AUCs of the clinical model and the integrative model validated by the validation set were 0.7808 (0.6231–0.9384) and 0.8462 (0.7132–0.9791). The quantitative radiomics signatures showed potential value in predicting the *EGFR* mutant and TMB status in GGOs. Moreover, the integrative model provided sufficient information for the selection of therapy and deserves further analysis.

## 1. Introduction

The increasing adhibition of low-dose CT-guided lung cancer screening and the use of the high-resolution diagnostic CT scan brought a sharp increase in the diagnoses of pulmonary nodules (PNs) [1,2]. Furthermore, about 40% of the PNs are known to be malignant, particularly in those in a high-risk population and having the ground-glass opacities (GGOs) of >10 mm in diameter [3,4]. A considerable proportion of patients were diagnosed with multiple GGOs [5], which were also classified as synchronous multiple primary lung cancer (sMPLC). However, patients with unresectable sMPLC remain a big challenge for surgeons, although surgery is usually the first selection for high-risk GGOs [6,7]. More than 70% of patients with lung cancer have locally advanced or distantly metastatic disease at the time of diagnosis [8], and the efficacy of the first-line chemotherapy is only approximately 30% [9].

In the past decade, tyrosine kinase inhibitors (TKIs) and immune checkpoint inhibitors (ICIs) have revolutionized the therapeutic landscape in lung cancers [10,11,12]. The effective rate of treatment with TKI in patients with EGFR-sensitive mutations is up to 70% [13], and EGFR-TKI are the main treatments for advanced lung adenocarcinoma (LUAD). Recently, neoadjuvant therapy using the PD-1 antibody [14,15], as well as EGFR-TKI [16] also exhibits potential prospects in patients with sMPLC. Individual treatments are based on patients’ clinic–pathologic characteristics, the tumor’s size and stage, individual somatic mutation status like *EGFR*, and the tumor’s mutation burden (TMB) status. TMB has attracted increasing attention due to its effective performance in predicting the response to PD-1 blockade immunotherapy in non-small cell lung cancer (NSCLC) and other solid tumors [12,17]. Several studies have also demonstrated that TMB^high^ status predicts a better prognosis for patients with resectable NSCLC [18]. Therefore, predicting individual molecular information, including TMB and somatic mutation, is meaningful for therapeutic strategies in early-stage lung cancer patients.

High-dimensional and quantitative radiomic features extracted from radiological images have shown promise in the prediction of diagnosis, prognosis, and optimal therapy of patients suffering from GGOs or lung cancer [19,20,21,22,23]. Previously, we established an efficient prediction model that predicts TMB status and *EGFR*/*TP53* mutations of early-stage LUAD, using the radiomics feature combined with the clinical information of 61 pulmonary nodules (PNs) from 51 LUAD patients [24]. However, as we were limited by the sample size, we obtained a perdition model with a relatively low AUC performance at only about 0.7. In the present study, we not only increased the sample size, but also tried a variety of statistical methods and selected the most appropriate one. Moreover, in order to predict the TMB status and *EGFR* mutations in patients with malignant PNs, we established an efficient CT-based radiomics model with specific clinical and radiomics features by dynamic nomogram and obtained a better prediction performance. We present the following article/case in accordance with the TRIPOD reporting checklist.

## 2. Methods

### 2.1. Study Population

Between January 2019 and December 2020, 93 patients with 108 GGOs were selected for analysis. The following inclusion criteria were used: (1) The maximum diameter of the nodule was less than 3 cm; (2) Next generation sequencing (NGS) tests and preoperative thin-section CT images were available; (3) the lesions can be seen on at least two consecutive layers of CT images; (4) there is a pathological diagnosis of lung adenocarcinoma; and (5) no antitumor therapy was received before surgery. This study was approved by the ethics committee at Jiangsu Cancer Hospital (Approval No. 2016 (220)) and complied with the Declaration of Helsinki. All participants provided written informed consent. NGS sequencing data and preoperative thin-section CT images were available from the database of the JSCH biobank. Clinical data collected for analysis was conducted within 1 week from the date of CT image acquisition, including age at diagnosis, gender, smoking status, BP/SP, blood types, biochemistry indicators and tumor markers. Smoking status was categorized into never smokers and smokers, and smokers included former or current smokers. In the step of data preprocessing, we considered the missing rate for each variable. Firstly, in the mutation of EGFR, TMB and radiomics variables are not missing. Secondly, we deleted nine clinical variables (including UALB, UGA, CA125, NSE, CA153, PCT, RDW.CV, CA199 and D.Dimer) with the missing rate larger than 20% (Supplementary Table S1). Finally, we used HotDeck to impute the remain 67 clinical variables. In the model we regarded gender, age and BMI and TMB as independent and dependent variables, respectively.

### 2.2. CT Image and 3D Reconstruction

All patients underwent pretreatment high-resolution CT scans to assure accurate volumetric analysis. The total nodule volume and GGO components of each lesion were determined by 3D reconstructions, and were automatically obtained using the Discovery CT750 HD scanner (GE Medical Systems, Milwaukee, WI, USA).

### 2.3. Tumor Segmentation and Radiomics Feature Extraction

**Figure 1:**
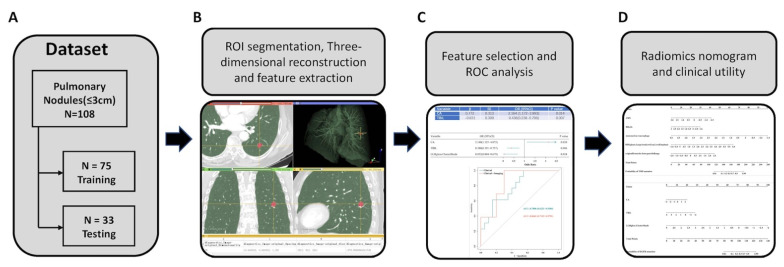
Study workflow overview. (**A**) Patient’s enrollment. (**B**) Region of interest (ROI) segmentation of pulmonary nodules and acquisition of radiomic features. (**C**) Feature selection and performance of the receiver operating characteristic (ROC) curve. (**D**) Performance of the radiomics nomogram and clinical utility.

As shown in Figure 1, CT images were imported into the 3D-Slicer 4.7.0 software (Harvard, MA, USA) and then contoured manually by three independent observers using the built-in paint tool. The delineation was performed in lung window setting (mean, −530~−430 HU; width, 1400~1600 HU) and then contoured manually by three independent observers using the built-in paint tool. Consensus was reached by discussion if there was interobserver variability.

Next, radiomics features were performed using a Radiomics plugin for the 3DSlicer [25]. All CT voxels were resampled to 1 mm^3^ for normalization using a cubic interpolation. In order to increase sensitivity relative to the original image, reduce image noise and normalize the intensities across all patients, we used a bin width of 25 Hounsfield units to discretize the intensities in the original image. In total, 870 radiomic features were extracted from the CT images of each patient, including the covering tumor intensity, shapes, wavelets, textures, and Gabor features [26]. All of the features defined in this package are in compliance with the feature definitions described by the Imaging Biomarker Standardization Initiative (IBSI), which are available in a separate document by A Zwanenburg, S Leger, M Vallières et al. [27].

### 2.4. Genomic Mutation Data Processing

The TMB and *EGFR* mutation data were obtained from the database of the JSCH biobank, as previously described [24]. Formalin-fixed paraffin-embedded (FFPE) malignant GGO samples were sliced and genomic DNA data was isolated from the slices. We conducted commercial pan-cancer panels on the Hiseq NGS platforms (Illumina Inc., San Diego, CA, USA). The definition of TMB is the rate of peptide changing single nucleotide variations (SNVs) per Mb, and TMB status is also the same as the previous study [24] in which >4 is relatively high (TMB^high^) and ≤4 is low (TMB^low^) [28].

## 3. Statistical Analysis

According to the ratio of 7:3, all patients were randomly assigned to the training set and the validation set. For the demographic characteristics, clinical characteristics and imaging parameters of patients, continuous variables were expressed by means ± SD, and categorical variables were described by percentages. Student’s *t* test was performed to compare the differences of the continuous variables, and Chi-square test was used to compare the distribution of the categorical variables between training set and validation set. Univariable logistic regressions were conducted to preliminarily select variables associated with the *EGFR* mutation and TMB status in the training set. Next, variables with *p* < 0.05 in univariable logistic regressions as candidate variables were included in LASSO (least absolute shrinkage and selection operator) regressions, which were performed 50 times to screen important variables among clinical characteristics and imaging parameters, respectively. Notably, before including lasso regressions, continuous variables were normalized. Then, clinical characteristics and imaging parameters, which were selected more than 25 times (frequency > 25) among 50 times lasso regressions, were included in the clinical model and imaging model, respectively. Meanwhile, stepwise binary logistic regressions were used to build the clinical model (only including clinical characteristics) and the integrative model (including clinical characteristics and imaging parameters). Finally, the receiver operator characteristic curve (ROC) was plotted, and its cutoff, sensitivity, specificity, positive predictive value and negative predictive value were calculated to evaluate the clinical model and the integrative model. In addition, nomograms were plotted to visualize two integrative models of *EGFR* mutations and TMB status.

Statistical analysis and figures were completed by using R software (Version 4.0.3, Vienna, Austria) and packages “compareGroups (Version 4.5.1)”, “glmnet (Version 4.1.3)”, “ggplot2 (Version 3.3.5)”, “forestplot (Version 2.0.1)”, “pROC (Version 1.18.0)” and “rms (Version 6.2-0)”. α = 0.05 was considered statistically significant.

## 4. Results

### 4.1. Patient Cohorts

We performed this study according to the Declaration of Helsinki. All patients signed the informed consent. This study was also approved by the Ethics Committee of the Jiangsu Cancer Hospital. The mean age was 57.82 ± 8.94 years, 30.56% was male and 12.96% smoked. The body mass index (BMI) was 23.14 ± 2.73 kg/m^2^, the mean arterial pressure (MAP) was 92.85 ± 9.38 mmHg. There were 55 (50.93%) patients with EGFR mutations and 49 (45.37%) patients with TMB-high status, respectively. According to the ratio 7:3, 108 patients were randomly assigned to the training cohort (75 patients, 69.44%) and the validation cohort (33 patients, 30.56%). The difference or distribution of characteristics were not significant between the training set and the validation set (all *p* values > 0.05). The details are shown in Table 1.

### 4.2. Prediction Model Construction for EGFR Mutations

**Figure 2:**
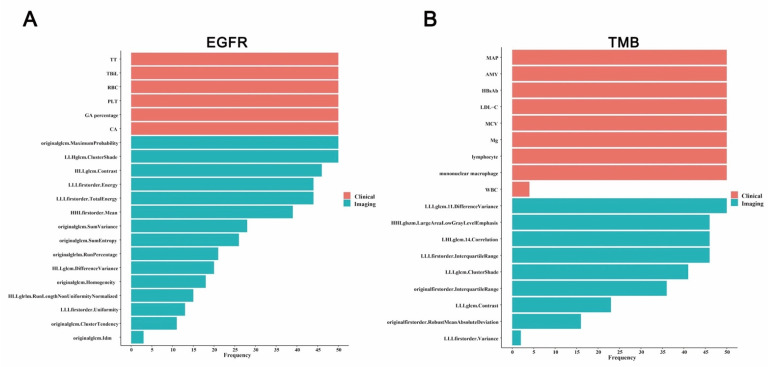
The importance of selected variables for *EGFR* mutations (**A**) and TMB status (**B**) prediction model. The importance means the variable frequency counted among 50 times lasso regressions.

**Figure 3:**
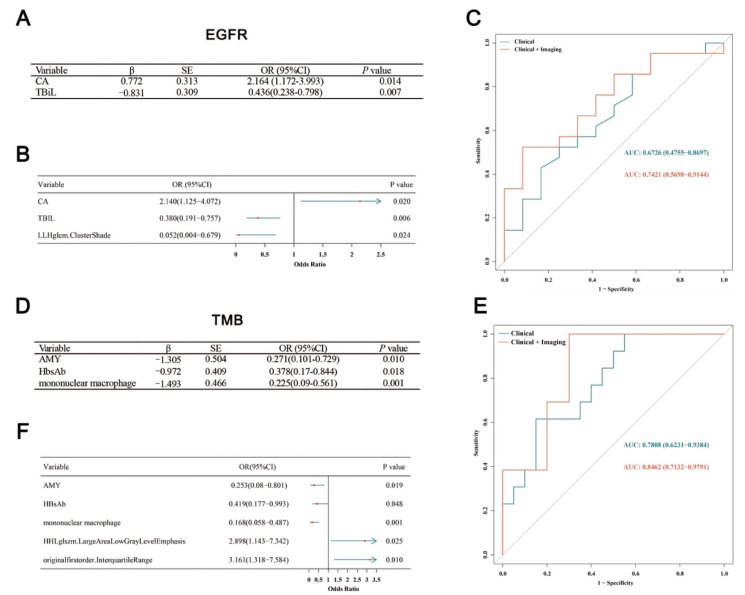
Selected variables information and prediction performance for the *EGFR* mutations and TMB status integrated model. (**A**,**D**) show selected variables of *EGFR* mutations and TMB status model. (**B**,**E**) show forest plots of selected variables of *EGFR* mutations and TMB status model. (**C**,**F**) show ROC of *EGFR* mutations and TMB status model.

In the first stage, 75 variables, including 8 clinical characteristics and 67 imaging parameters, were statistically associated with EGFR mutations identified by univariable logistic regressions (all *p* < 0.05), as listed in Table S2. In the 2th stage, the above variables were included in 50 times lasso regressions with family “binomial” for EGFR mutations. The variable (Thrombin time (TT), Total bilirubin (TBiL), Red blood cell (RBC), Platelet count (PLT), Glycated albumin (GA) percentag, Carbohydrate antigen (CA), originalglem.MaximumProbability, LLHglcm.ClusterShade, HLLglcm.Contrast, LLLfirstorder.Energy, LLLfirstorder.TotalEnergy, etc.) and their frequency selected among 50 times lasso regressions were visualized using the bar plot, as shown in Figure 2A. In the 3th stage, the clinical model (Figure 3A) built by stepwise logistic regression demonstrated that CA (OR = 2.164, 95%CI: 1.172–3.993, *p* = 0.014) and TBiL (OR = 0.436, 95%CI: 0.238–0.798, *p* = 0.007) were the predictors of EGFR mutations, and the integrative model (Figure 3B) suggested that a radiomic wavelet feature, LLHglcm.ClusterShade (OR = 0.052, 95%CI: 0.004–0.679, *p* = 0.024), was a predictor of EGFR mutations as well as CA (OR = 2.140, 95%CI:1.125–4.072, *p* = 0.020) and TBiL (OR = 0.380, 95%CI: 0.191–0.757, *p* = 0.006).

We next validated the predictive effects in the test set. For *EGFR* mutations, the AUCs (Figure 3C) of clinical and integrative models were 0.6726 (0.4755–0.8697) and 0.7421 (0.5698–0.9144), respectively. AUCs of the integrative models for the *EGFR* mutation, including imaging parameters, were larger than that of the clinical-only models, which means that the discrimination (AUCs) of the integrative model was better. The specificity and positive predictive value of the integrated model for *EGFR* mutations were both 0.917, which means that the ability of the integrative model to identify and exclude non-mutation was strong, and the proportion of patients who did have the mutation was higher among those with the mutation found by the model.

### 4.3. Prediction Model Construction for TMB Status

In the first stage, 59 variables, 11 clinical characteristics and 48 imaging parameters were statistically associated with TMB status identified by univariable logistic regressions (all *p* < 0.05), as listed in Table S3. In the second stage, the above variables were included in 50 times lasso regressions. The variable (Mean arterial pressure (MAP), Amylase (AMY), Hepatitis B surface antibody (HBsAb), Low-density lipoprotein cholesterol (LDL-C), Mean corpusular volume (MCV), Magnesium (Mg), lymphocyte, mononuclear macrophage, LLLglcm.DifferenceVariance, HHLglszm.LargeAreaLowGrayLevelEmphasis, LHLglcm.Correlation, LLLfirstorder.InterquartileRange, etc.) and their frequency selected among 50 times lasso regressions were visualized using the bar plot, as shown in Figure 2B. In the third stage, the clinical model (Figure 3D) for predicting TMB status showed that AMY (OR = 0.271, 95%CI: 0.101–0.729, *p* = 0.010), HbsAb (OR = 0.378, 95%CI: 0.17–0.844, *p* = 0.018) and mononuclear macrophage (OR = 0.225, 95%CI: 0.09–0.561, *p* = 0.001) were predictors, and two radiomic features, HHLglszm.LargeAreaLowGrayLevel (OR = 2.898, 95%CI: 1.143–7.342, *p* = 0.025) and originalfirstorder.InterquartileRange (OR = 3.161, 95%CI: 1.318–7.584, *p* =0.010) were also predictors in the integrative model for TMB status (Figure 3E). Next, with regards to TMB status, the AUCs of two models (Figure 3F) were 0.7808 (0.6231–0.9384) and 0.8462 (0.7132–0.9791), respectively. It could be also seen from the above that AUCs of the integrative models for TMB status were larger than that of clinical models. Meanwhile, the sensitivity and negative predictive value of the integrative model for TMB status were both 1.000, which means that the integrative model could fully identify the TMB status of all patients.

### 4.4. Decision Curve Analysis

**Figure 4:**
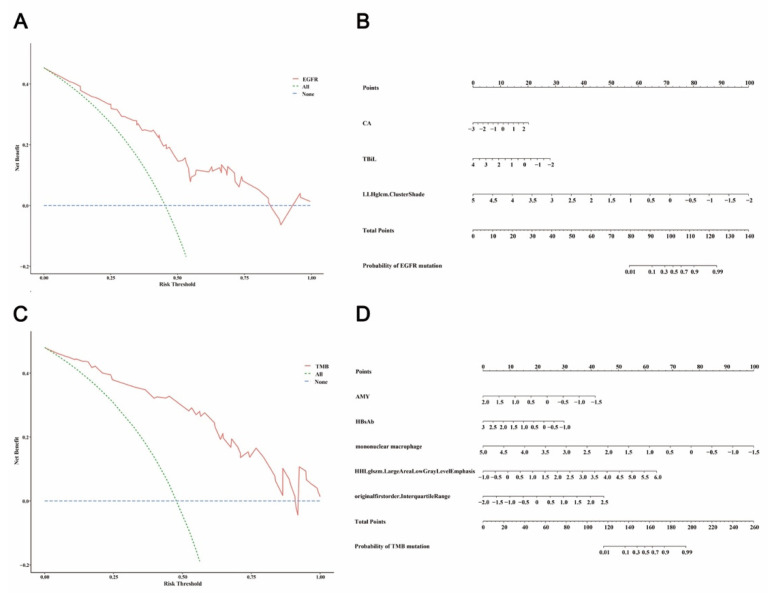
The nomogram and decision curve of the integrated prediction model for *EGFR* mutations (**A**,**B**) and TMB status (**C**,**D**).

Decision curves [29] of predictive models for the *EGFR* mutation and TMB status were plotted, as showed in Figure 4. For the *EGFR* mutation, risk-based interventions based on the integrated model is recommended when the risk threshold is between 20% and 80% (Figure 4A). For TMB status, risk-based interventions based on the integrated model is recommended when the risk threshold is between 10% and 90% (Figure 4B).

### 4.5. Nomograms for Predicting EGFR Mutation and TMB Status

Finally, two validated integrative models were visualized as the nomogram in the study (Figure 4), which could be used to predict individual risk and guide individualized treatment. In other words, we can calculate the total point according to the standardized variable value and the corresponding point. Next, we could obtain the mutation probability of certain patients. For instance, in Figure 4A, the total point of patients whose CA = 2.5 (Point ≈ 20), TBiL = 2 (Point ≈ 30) and LLHglcm.ClusterShade = 1 (Point ≈ 58) were about 108. Therefore, the probability of the patient harboring *EGFR* mutations was more than 70%. In addition, two web-based dynamic nomograms for EGFR mutations (https://ww-jshtcm.shinyapps.io/Dynamic_nomogram_EGFR/ accessed on 9 November 2022) and TMB status (https://ww-jshtcm.shinyapps.io/Dynamic_nomogram_TMB/ accessed on 9 November 2022) were deployed on the website.

## 5. Discussion

There are usually no typical signs or symptoms of lung cancer in the early stages. The now wide use of low-dose CT in lung cancer diagnosis has led to a considerable number of patients being diagnosed with sMPLC. Jing L, Dong Z, Xiao W et al. [30] conducted a retrospective analysis of 164 patients and found that the overall survival and progression-free survival rates with sMPLC were 72.6% and 61.0%, respectively. Kocaturk CI, Gunluoglu MZ, Cansever L et al. [31] reported that the 5-year survival rate was 40.6% for unilateral and 62.8% for bilateral sMPLC patients who received the surgical resection. The revolutionary effects of TKI and ICI treatment on lung cancer brings new hope to those patients. Therefore, it is of great importance to predict the TMB and EGFR status of patients with sMPLC.

In the present study, we construct a prediction model from the training cohort (75 PNs) and evaluated the performance of the model in an independent validation cohort (33 PNs). For *EGFR* mutations, the AUCs of clinical and integrative models were 0.6726 (0.4755–0.8697) and 0.7421 (0.5698–0.9144), respectively. For the TMB status, the AUCs of two models were 0.7808 (0.6231–0.9384) and 0.8462 (0.7132–0.9791), respectively. Compared with our former study [24], we increased the sample size and improve the statistical methods, obtained an efficient CT-based radiomics model and better prediction performance. The prediction model revealed that there was a significant association between CT features, *EGFR* mutation and TMB status. Our works provide a non-invasive method to assess *EGFR* and TMB information for patients, and offers an alternative supplement to biopsy.

Previously, studies focused on predicting the *EFGR* mutation and TMB status used clinical factors and radiomics based on feature engineering such as gender, age, tumor stage and predominant subtype [32,33]. Obviously, clinical features can only reflect tumor information, partly on a pathological level. Radiomics studies can quantify medical figures into image features, and identify the connections between these features and gene characteristics by feature selection, statistical analysis and other methods to characterize the phenotype of the tumors and clinical utility [20]. Wen Q, et al. [21] showed that radiomics signatures demonstrated a positive performance for predicting PD-L1 and TMB with AUCs of 0.730 and 0.759, respectively. The model that combined radiomics signatures with clinical and morphological factors has improved the predictive efficacy reached for PD-L1 (AUC = 0.839) and TMB (*p* = 0.818). We have also harbored better recognition ability (0.7421 for *EGFR* and 0.8462 for TMB). Moreover, the positive predictive value of the integrated model for *EGFR* mutations was 0.917, which indicates that the ability of the integrative model to identify the *EGFR* mutation and nodules’ benefit from cancer genetic testing was strong. At the same time, the negative predictive value of the integrative model for TMB status was 1.000, which helps clinicians reduce unnecessary tests. Based on the different purpose of the integrative model that was achieved, we therefore have reason to believe that the model is practical, and we will be able to gain higher accuracy if multi-center cooperation is established in the future.

Despite these encouraging results, this study does have some limitations. Firstly, this study was a single-institutional and small-sample study, therefore we will construct a multi-institutional and larger sample study in the future. Secondly, we conducted a retrospective study, which may bring potential bias to the results of the study. In future studies, we will prospectively apply our radiological characteristics to clinical practice, which is also an important part of the pre-treatment evaluation. Thirdly, the image texture features in our study were extracted from the data via manual segmentation by several experienced imaging doctors; it was difficult to exclude the small blood vessels and bronchus in the nodule, which may affect the accuracy of some features. Fourthly, the other driver mutations such as *ALK* and *TP53*, and their correlation with the features within the radiomics signature was not explored. Lastly, all of the patients included in this study had malignant PNs (<3 cm), which limits the use of this method in patients with advanced disease. We will therefore include advanced lung cancer patients in our future work to increase the sample size.

In conclusion, our present study shows that the quantitative radiomics features extracted from CT images were non-invasively associated with *EGFR* and TMB status. The integrated model built by radiomics features combined with clinical factors that significantly improved the predictive performance, which is of great help for physicians to make effective clinical plans.

## Figures and Tables

**Table 1:** Characteristics of patients.

Characteristic	All *n* = 108	Data Set		*p* Value
		Train (*n* = 75)	Validation (*n* = 33)	
Age, year	57.82 ± 8.94	57.84 ± 8.48	57.79 ± 10.03	0.979
Sex, %				0.104
Male	33 (30.56)	27 (36.00)	6 (18.18)	
Female	75 (69.44)	48 (64.00)	27 (81.82)	
Smoking, %				0.544
No	94 (87.04)	64 (85.33)	30 (90.91)	
Yes	14 (12.96)	11 (14.67)	3 (9.09)	
*BMI, kg/m^2^	23.14 ± 2.73	23.31 ± 2.91	22.75 ± 2.27	0.285
*MAP, mmHg	92.85 ± 9.38	91.96 ± 9.80	94.86 ± 8.12	0.114
*EGFR mutation, %				0.123
No	53 (49.07)	41 (54.67)	12 (36.36)	
Yes	55 (50.93)	34 (45.33)	21 (63.64)	
*TMB, %				0.537
No	59 (54.63)	39 (52.00)	20 (60.61)	
Yes	49 (45.37)	36 (48.00)	13 (39.39)	

*BMI, Body mass index; *MAP, Mean arterial pressure; *EGFR, Epidermal growth factor receptor; *TMB, Tumor mutation burden

## Data Availability

The data supporting the findings of the present study are available within the paper and its supplementary information files. All other relevant deidentified data related to the present study are available from the corresponding author (Rong Yin) upon reasonable academic request. Source data are provided with this paper.

## Supplementary Materials

The following are available online at https://www.mdpi.com/article/10.3390/jpm13010016/s1, Table S1: Clinical variables and missing rate; Table S2: Variables associated with *EGFR* mutations in univariable logistic regressions; Table S3: Variables associated with TMB mutations in univariable logistic regressions.

## Abbreviations

AMY	Amylase
AUC	Area under curve
BMI	Body mass index
CA	Carbohydrate antigen
CT	Computed tomography
DCA	Decision curve analysis
DNA	Deoxyribonucleic acid
EGFR	Epidermal growth factor receptor
FPR	False positive rate
GA	Glycated albumin
GGO	Ground-glass opacity
HBsAb	Hepatitis B surface antibody
ICI	Immune checkpoint inhibitors
LASSO	Least absolute shrinkage and selection operator
LDL-C	Low-density lipoprotein cholesterol
LUAD	Lung adenocarcinoma
MAP	Mean arterial pressure
MCV	Mean corpusular volume
Mg	Magnesium
NGS	Next-generation sequencing
NSCLC	Non-small cell lung cancer
NSE	Neuron specific enolase
OS	Overall survival
PCT	Plateletocrit
PD-1	Programed death-1
PLT	Platelet count
PN	Pulmonary nodule
RBC	Red blood cell
RDW	Red cell volume distribution width
ROC	Receiver operating characteristic curve
ROI	Region of interest
sMPLC	Synchronous multiple primary lung cancer
SABR	Stereotactic ablative radiotherapy
TBiL	Total bilirubin
TKI	Tyrosine kinase inhibitors
TMB	Tumor mutation burden
TPR	True positive rate
TT	Thrombin time
UGA	Urine glucaric acid
UALB	Urinary albumin
