# Supplementary material for: Predicting Tumor Mutation Burden and EGFR Mutation Using Clinical and Radiomic Features in Patients with Malignant Pulmonary Nodules
Source: J Pers Med. 2022 Dec 22;13(1):16. doi: 10.3390/jpm13010016 (PMC9865229; doi:10.3390/jpm13010016)
Supplement: Supplementary file 1 [file jpm-13-00016-s001.zip › jpm-2055026-supplementary.pdf]

Table S1 Clinical variables and missing rate

Feature	MissRate	Feature	MissRate	Feature	MissRate	Feature	MissRate
ALB	0.564815	HCV	0.037037	CO2	0	MCHC	0
UGA	0.564815	MPV	0.018519	CREA	0	MCV	0
CA125	0.472222	PDW	0.018519	DBIL	0	Mg	0
NSE	0.444444	Age	0	E0S.	0	MON.	0
CA153	0.435185	Gender	0	EOS.	0	MON..1	0
PCT	0.333333	MAP	0	FIB	0	Na	0
RDW.CV	0.333333	BMI	0	GGT	0	NEU.	0
CA199	0.277778	AG	0	GLOB	0	NEU..1	0
D.Dimer	0.240741	ALB	0	GLU	0	P	0
GA.	0.157407	ALP	0	HCT	0	PLT	0
P.LCR	0.064815	ALT	0	HDL.C	0	PT	0
SBP	0.046296	AMY	0	HGB	0	RBC	0
DBP	0.046296	APTT	0	INR	0	TBIL	0
Smoke	0.037037	AST	0	K	0	TG	0
CEA	0.037037	BAS.	0	LDH	0	TP	0
HBcAb	0.037037	BAS..1	0	LDL.C	0	TT	0
HBeAb	0.037037	CA	0	LYM.	0	UA	0
HBeAg	0.037037	CHOL	0	LYM..1	0	UREA	0
HBsAb	0.037037	CL	0	MCH	0	WBC	0

Table S2 Variables associated with *EGFR* mutations in univariable logistic regressions

Variable	β	SE	OR(95%CI)	*P* value
CA	0.796	0.239	2.217(1.389-3.539)	<0.001
DBiL	-0.530	0.220	0.589(0.382-0.907)	0.016
GA%	0.599	0.227	1.820(1.168-2.839)	0.008
HCT	0.431	0.210	1.539(1.019-2.321)	0.040
PLT	0.522	0.213	1.685(1.109-2.560)	0.015
RBC	0.454	0.206	1.575(1.051-2.36)	0.028
TBiL	-0.516	0.217	0.597(0.39-0.913)	0.017
TT	0.526	0.213	1.692(1.114-2.572)	0.014
originalshape.Compactness2	-0.448	0.206	0.639(0.427-0.957)	0.030
originalshape.Sphericity	-0.501	0.209	0.606(0.402-0.913)	0.017
originalshape.Compactness1	-0.489	0.208	0.613(0.408-0.921)	0.019
originalshape.SphericalDisproportion	0.533	0.218	1.704(1.112-2.612)	0.014
originalfirstorder.InterquartileRange	0.428	0.207	1.534(1.022-2.303)	0.039
originalfirstorder.Uniformity	-0.733	0.259	0.48(0.289-0.798)	0.005
originalfirstorder.Energy	0.405	0.204	1.499(1.005-2.236)	0.047
originalfirstorder.RobustMeanAbsoluteDeviation	0.404	0.206	1.498(1.001-2.241)	0.049
originalfirstorder.TotalEnergy	0.405	0.204	1.499(1.005-2.236)	0.047
originalfirstorder.Entropy	0.633	0.219	1.883(1.226-2.894)	0.004
originalglcm.SumVariance	0.478	0.223	1.613(1.041-2.498)	0.033

originalglcm.Homogeneity1	-0.567	0.224	0.567(0.366-0.879)	0.011
originalglcm.Homogeneity2	-0.563	0.225	0.569(0.366-0.885)	0.012
originalglcm.MaximumProbability	-0.697	0.280	0.498(0.288-0.863)	0.013
originalglcm.Contrast	0.443	0.211	1.557(1.029-2.357)	0.036
originalglcm.DifferenceEntropy	0.568	0.214	1.765(1.159-2.684)	0.008
originalglcm.InverseVariance	-0.478	0.206	0.62(0.414-0.929)	0.021
originalglcm.Dissimilarity	0.502	0.211	1.652(1.093-2.498)	0.017
originalglcm.Idm	-0.563	0.225	0.569(0.366-0.885)	0.012
originalglcm.SumEntropy	0.578	0.215	1.782(1.170-2.718)	0.007
originalglcm.Energy	-0.784	0.318	0.457(0.245-0.851)	0.014
originalglcm.SumSquares	0.486	0.222	1.626(1.052-2.512)	0.028
originalglcm.Entropy	0.639	0.219	1.895(1.234-2.909)	0.004
originalglcm.DifferenceAverage	0.502	0.211	1.652(1.093-2.498)	0.017
originalglcm.Id	-0.567	0.224	0.567(0.366-0.879)	0.011
originalglcm.ClusterTendency	0.478	0.223	1.613(1.041-2.498)	0.032
originalglrlm.GrayLevelNonUniformityNormalized	-0.644	0.231	0.525(0.334-0.826)	0.005
originalglrlm.RunVariance	-0.601	0.303	0.548(0.303-0.993)	0.047
originalglrlm.LongRunEmphasis	-0.595	0.291	0.552(0.312-0.975)	0.041
originalglrlm.ShortRunEmphasis	0.508	0.233	1.662(1.054-2.623)	0.029
originalglrlm.RunPercentage	0.509	0.236	1.664(1.048-2.639)	0.031
originalglrlm.RunLengthNonUniformityNormalized	0.481	0.222	1.618(1.047-2.501)	0.030
originalglszm.ZoneEntropy	0.432	0.209	1.540(1.021-2.321)	0.039
LHLfirstorder.Skewness	-0.434	0.210	0.648(0.43-0.977)	0.038
LHLglszm.SmallAreaLowGrayLevelEmphasis	-0.442	0.216	0.643(0.421-0.981)	0.040
HLLfirstorder.Entropy	0.465	0.208	1.592(1.059-2.393)	0.025
HLLfirstorder.10Percentile	-0.451	0.215	0.637(0.418-0.971)	0.036
HLLglcm.SumVariance	0.504	0.232	1.655(1.051-2.607)	0.030
HLLglcm.Homogeneity1	-0.487	0.208	0.614(0.409-0.925)	0.019
HLLglcm.Homogeneity2	-0.473	0.208	0.623(0.415-0.936)	0.023
HLLglcm.Contrast	0.575	0.242	1.777(1.106-2.856)	0.017
HLLglcm.DifferenceEntropy	0.481	0.208	1.618(1.077-2.432)	0.021
HLLglcm.InverseVariance	-0.413	0.203	0.662(0.444-0.985)	0.042
HLLglcm.Dissimilarity	0.534	0.219	1.706(1.112-2.618)	0.015
HLLglcm.DifferenceVariance	0.523	0.236	1.687(1.063-2.678)	0.027
HLLglcm.Idm	-0.473	0.208	0.623(0.415-0.936)	0.023
HLLglcm.SumEntropy	0.463	0.207	1.589(1.058-2.385)	0.026
HLLglcm.SumSquares	0.534	0.236	1.706(1.073-2.711)	0.024
HLLglcm.Entropy	0.514	0.210	1.672(1.107-2.523)	0.015
HLLglcm.DifferenceAverage	0.534	0.219	1.706(1.112-2.618)	0.015
HLLglcm.Id	-0.487	0.208	0.614(0.409-0.925)	0.019
HLLglcm.ClusterTendency	0.504	0.232	1.655(1.051-2.607)	0.030
HLLglrlm.GrayLevelVariance	0.494	0.232	1.639(1.039-2.583)	0.034
HLLglrlm.ShortRunEmphasis	0.425	0.209	1.530(1.015-2.304)	0.042
HLLglrlm.RunPercentage	0.420	0.210	1.522(1.009-2.295)	0.045

HLLglrlm.RunLengthNonUniformityNormalized	0.434	0.207	1.543(1.028-2.317)	0.036
LLHglcm.ClusterShade	-1.754	0.743	0.173(0.04-0.742)	0.018
HHLfirstorder.Mean	0.470	0.239	1.600(1.002-2.554)	0.049
LLLfirstorder.Uniformity	-0.429	0.212	0.651(0.43-0.987)	0.043
LLLfirstorder.Energy	0.653	0.318	1.921(1.031-3.581)	0.040
LLLfirstorder.TotalEnergy	0.653	0.318	1.921(1.031-3.581)	0.040
LLLglcm.Homogeneity1	-0.426	0.210	0.653(0.432-0.986)	0.043
LLLglcm.Homogeneity2	-0.441	0.217	0.643(0.42-0.984)	0.042
LLLglcm.MaximumProbability	-0.502	0.227	0.605(0.388-0.944)	0.027
LLLglcm.InverseVariance	-0.411	0.209	0.663(0.440-1.000)	0.050
LLLglcm.Idm	-0.441	0.217	0.643(0.42-0.984)	0.042
LLLglcm.Id	-0.426	0.210	0.653(0.432-0.986)	0.043

Table S3 Variables associated with TMB mutations in univariable logistic regressions

Variable	β	SE	OR(95%CI)	P value
SBP	-0.033	0.016	0.968(0.938-0.998)	0.039
MAP	-0.435	0.211	0.647(0.428-0.980)	0.040
AMY	-1.005	0.308	0.366(0.200-0.670)	0.001
HBsAb	-0.723	0.267	0.485(0.287-0.819)	0.007
LDLC	-0.502	0.210	0.605(0.401-0.914)	0.017
LYM count	-1.199	0.401	0.301(0.137-0.661)	0.003
MCH	0.484	0.239	1.623(1.014-2.593)	0.043
MCV	0.545	0.246	1.725(1.066-2.790)	0.026
Mg	-0.435	0.205	0.647(0.433-0.968)	0.034
MON_count	-1.208	0.343	0.299(0.152-0.586)	<0.001
WBC	-1.301	0.549	0.272(0.093-0.798)	0.018
originalfirstorder.InterquartileRange	0.522	0.211	1.685(1.115-2.548)	0.013
originalfirstorder.MeanAbsoluteDeviation	0.554	0.212	1.740(1.149-2.636)	0.009
originalfirstorder.RobustMeanAbsoluteDeviation	0.563	0.213	1.756(1.156-2.665)	0.008
originalfirstorder.Median	0.393	0.200	1.481(1.00-2.195)	0.050
originalfirstorder.90Percentile	0.449	0.202	1.567(1.055-2.328)	0.026
originalfirstorder.StandardDeviation	0.530	0.21	1.699(1.124-2.566)	0.017
originalfirstorder.Variance	0.573	0.217	1.774(1.159-2.715)	0.008
originalglcm.SumVariance	0.561	0.227	1.752(1.124-2.731)	0.013
originalglcm.ClusterShade	-0.523	0.232	0.593(0.376-0.934)	0.024
originalglcm.SumAverage	0.505	0.216	1.657(1.084-2.532)	0.020
originalglcm.DifferenceVariance	0.510	0.211	1.665(1.100-2.520)	0.016
originalglcm.Autocorrelation	0.582	0.267	1.790(1.060-3.020)	0.029
originalglcm.AverageIntensity	0.505	0.216	1.657(1.084-2.532)	0.020
originalglcm.SumSquares	0.540	0.222	1.716(1.110-2.653)	0.015
originalglcm.ClusterProminence	0.688	0.277	1.990(1.155-3.425)	0.013
originalglcm.ClusterTendency	0.561	0.227	1.752(1.124-2.731)	0.013
originalglrlm.GrayLevelVariance	0.621	0.227	1.861(1.193-2.903)	0.006
originalglrlm.ShortRunHighGrayLevelEmphasis	0.622	0.277	1.863(1.082-3.206)	0.025
originalglrlm.HighGrayLevelRunEmphasis	0.564	0.272	1.758(1.032-2.996)	0.038
originalglszm.GrayLevelVariance	0.444	0.216	1.559(1.02-2.384)	0.040
originalglszm.SizeZoneNonUniformityNormalized	0.522	0.209	1.685(1.119-2.540)	0.013
originalglszm.SmallAreaEmphasis	0.521	0.209	1.684(1.118-2.537)	0.013
LHLglcm.Correlation	0.491	0.21	1.634(1.084-2.465)	0.019
HHLglszm.LargeAreaLowGrayLevelEmphasis	0.465	0.235	1.592(1.004-2.524)	0.048
LLLfirstorder.InterquartileRange	0.587	0.216	1.799(1.177-2.749)	0.007
LLLfirstorder.MeanAbsoluteDeviation	0.585	0.214	1.795(1.179-2.732)	0.006
LLLfirstorder.RobustMeanAbsoluteDeviation	0.601	0.217	1.824(1.192-2.788)	0.006
LLLfirstorder.Median	0.399	0.201	1.490(1.004-2.211)	0.047
LLLfirstorder.90Percentile	0.464	0.202	1.59(1.07-2.365)	0.022
LLLfirstorder.StandardDeviation	0.559	0.212	1.749(1.154-2.653)	0.008
LLLfirstorder.Variance	0.651	0.225	1.917(1.234-2.981)	0.004

LLLfirstorder.Mean	0.397	0.201	1.487(1.003-2.207)	0.048
LLLglcm.SumVariance	0.64	0.221	1.896(1.228-2.926)	0.004
LLLglcm.ClusterShade	-0.505	0.242	0.604(0.375-0.97)	0.037
LLLglcm.Contrast	0.499	0.213	1.647(1.084-2.502)	0.019
LLLglcm.SumAverage	0.519	0.219	1.68(1.094-2.579)	0.018
LLLglcm.DifferenceVariance	0.672	0.233	1.958(1.241-3.09)	0.004
LLLglcm.Autocorrelation	0.577	0.265	1.781(1.059-2.994)	0.029
LLLglcm.AverageIntensity	0.519	0.219	1.68(1.094-2.579)	0.018
LLLglcm.SumSquares	0.64	0.221	1.896(1.231-2.921)	0.004
LLLglcm.ClusterProminence	0.926	0.316	2.524(1.358-4.691)	0.003
LLLglcm.ClusterTendency	0.64	0.221	1.896(1.228-2.926)	0.004
LLLglrlm.GrayLevelVariance	0.691	0.225	1.996(1.285-3.101)	0.002
LLLglrlm.ShortRunHighGrayLevelEmphasis	0.584	0.266	1.793(1.064-3.022)	0.028
LLLglrlm.HighGrayLevelRunEmphasis	0.561	0.268	1.752(1.036-2.962)	0.036
LLLglszm.GrayLevelVariance	0.666	0.231	1.946(1.236-3.063)	0.004
LLLglszm.SmallAreaHighGrayLevelEmphasis	0.607	0.278	1.835(1.064-3.164)	0.029
LLLglszm.HighGrayLevelZoneEmphasis	0.571	0.271	1.770(1.041-3.009)	0.035
